# Multiscale Feature-Learning with a Unified Model for Hyperspectral Image Classification

**DOI:** 10.3390/s23177628

**Published:** 2023-09-03

**Authors:** Tahir Arshad, Junping Zhang, Inam Ullah, Yazeed Yasin Ghadi, Osama Alfarraj, Amr Gafar

**Affiliations:** 1 School of Electronics and Information Engineering, Harbin Institute of Technology, Harbin 150001, China; tas25@yahoo.com (T.A.); zhangjp@hit.edu.cn (J.Z.); 2 Department of Computer Engineering, Gachon University, Seongnam 13120, Republic of Korea; 3 Department of Computer Science, Al Ain University, Abu Dhabi P.O. Box 112612, United Arab Emirates; yazeed.ghadi@aau.ac.ae; 4 Computer Science Department, Community College, King Saud University, Riyadh 11437, Saudi Arabia; oalfarraj@ksu.edu.sa; 5 Mathematics and Computer Science Department, Faculty of Science, Menofia University, Shebin Elkom 6131567, Egypt; atolba@science.menofia.edu.eg

**Keywords:** feature extraction, multiscale features, deep learning models, hyperspectral image classification, convolutional neural network, swin transformer

## Abstract

In the realm of hyperspectral image classification, the pursuit of heightened accuracy and comprehensive feature extraction has led to the formulation of an advance architectural paradigm. This study proposed a model encapsulated within the framework of a unified model, which synergistically leverages the capabilities of three distinct branches: the swin transformer, convolutional neural network, and encoder–decoder. The main objective was to facilitate multiscale feature learning, a pivotal facet in hyperspectral image classification, with each branch specializing in unique facets of multiscale feature extraction. The swin transformer, recognized for its competence in distilling long-range dependencies, captures structural features across different scales; simultaneously, convolutional neural networks undertake localized feature extraction, engendering nuanced spatial information preservation. The encoder–decoder branch undertakes comprehensive analysis and reconstruction, fostering the assimilation of both multiscale spectral and spatial intricacies. To evaluate our approach, we conducted experiments on publicly available datasets and compared the results with state-of-the-art methods. Our proposed model obtains the best classification result compared to others. Specifically, overall accuracies of 96.87%, 98.48%, and 98.62% were obtained on the Xuzhou, Salinas, and LK datasets.

## 1. Introduction

Hyperspectral image data are acquired through hyperspectral sensors, capturing both spatial and spectral information from the visible to infrared spectrum for each pixel [1]. These images provide detailed spatial characteristics of objects along with their continuous diagnostic spectra [2]. Due to the valuable combination of multiscale spectral and spatial information, hyperspectral data find applications in various domains such as agriculture [3,4], mineralogy [5], earth observation [6], and other related applications [7,8,9]. How to classify hyperspectral images and extract multiscale features effectively is a hot topic for researchers. Various data processing techniques have been explored to effectively utilize acquired hyperspectral images, including unmixing, detection, and classification [10]. How to use hyperspectral image classification is also a hotspot topic. In previous studies, traditional machine learning algorithms were employed for HSI classification, including k-nearest neighbor [11], logistic regression [12], Bayesian estimation [13], and support vector machines [14]. However, it was observed that these conventional classification approaches often resulted in misclassification. In addition, several methods for dimensionality reduction and spectral information extraction have been developed, such as principle component analysis [15], independent component analysis [16], and linear discriminative analysis [17]. However, these methods tend to overlook the spatial correlation among pixels in a spatial dimension, which is crucial for optimal spatial feature extraction. To address this limitation, various mathematical operators have been developed, such as morphological profile [18], extended morphological operator [19], and extended multiattribute profile [20,21,22,23,24,25,26,27,28,29,30,31,32,33,34,35,36,37,38,39,40,41,42,43,44,45,46,47].

In recent years, deep learning models, especially convolutional neural networks, have shown significant advantages over traditional methods in extracting more relevant and discriminative multiscale features. In [21], a stacked autoencoder and deep belief network were applied to extract multiscale features. These methods required a 1D feature as input. In [22], a 2D-CNN was proposed to carry out principle component analysis after the dimensionality reduction process. In [23], a more effective method for extracting spatial–spectral features in 3D-CNNs was proposed. In [24], combining 3D and 2D-CNN characteristics to reduce the computational complexity and improve classification accuracy HybridCNN was proposed. In [25], the author developed two stream residual deep feature fusion convolutional neural networks to fuse two branches to extract multiscale spatial spectral features. One branch was used for global feature extraction, and the other branch was used for local feature extraction. Moreover, recurrent neural networks [26], generative adversarial neural networks [27], graph neural networks [28], and capsule networks were proposed [29]. Conversely, encoder–decoder architectures are often employed in unsupervised multiscale feature learning to extract and reconstruct features from hyperspectral images [43]. Nonetheless, it is widely acknowledged that deep learning methodologies demand a substantial amount of labeled samples and an extensive number of training epochs, posing significant challenges in hyperspectral image classification.

Recently, a new model vision transformer [30] has exhibited better performance in the domain of computer vision. The transformer uses a self-attention mechanism to extract global dependencies. Attention mechanisms are also widely used in HSI classification. In [31], the spectral–spatial attention network was designed to extract discriminative features from the HSI cube. Much work has been carried out to apply the vision transformer model to hyperspectral image classification. In [32], spectral–spatial transformers (SST) were proposed. The author used a similar VGGNet model for spectral and spatial feature extraction and developed a relationship with a dense transformer. In [33], the author proposed a new model called SpectralFormer. This model can learn GroupWise spectral information and design cross-layer transformer encoders. In [34], the author introduced a spectral–spatial feature tokenization transformer for HSI classification; it uses 3D and 2D-CNN models for multiscale spatial and spectral features, in addition to a Gaussian weighted tokenizer. In [35], the author used a convolution network with a transformer model called CT Mixer and introduced a novel local global multi-head self-attention. In [36], the author proposed two branches of pure transformers: one is the spectral branch, and the other is a spatial branch. For the spectral branch, the author used a vision transformer for spectral features and for the spatial branch, the author used a swin transformer for spatial features; at the end, branch fusion strategy was used to learn joint features. Inspired by [42], depending on the desired information, different types of features can be extracted, such as pixel-based and structure-based features. However, finding an efficient and universal approach to fuse these features optimally remains a challenge due to the subtle relationship between the data. 

In [48], the author proposed a model for deep multiscale feature learning for distorted image quality assessment. The author proposed a two-branch network for distorted images and residual maps. The network consists of spatial pyramid pooling and feature pyramid, aiming to learn hierarchical multiscale features from images. In addition, the author of [49] proposed DeepCervix, a hybrid deep feature fusion technique based on deep learning. In this method, various deep learning pre-trained models such as VGG16, VGG19, XceptionNet, and ResNet50 models are used to capture multiscale information to enhance the classification performance. In [50], the author proposed a multiscale feature fusion model based on ResNet50 and VGG16; they extract multiscale feature vectors of the last layer before the softmax layer of these two models. In [51], the author proposed the DeepFusion model to extract structural similarity features and sub-structure features and feed them into the interaction feature fusion module to encode interaction features. In [52], the author proposed a deep feature fusion classification network (DFFCNet) based on EfficientNetV2 as a backbone network ResNet with channel attention and a spatial attention module to fuse the features. These methods adopt concatenation or adding techniques to fuse the features of branches, causing an increase in dimensionality and an increase in computational cost. However, these methods perform well and obtain multiscale features with fusion techniques, but they are not capable of extracting high-level multiscale features.

Therefore, to take advantage of different models and their ability to extract multiscale features, we propose a swin transformer [34] with deep model architecture. To extract multiscale features, we propose a model that has three branches. First, we use a fully connected encoder–decoder with an attention module to reconstruct the spectral features. Secondly, we use a convolutional neural network to extract the multiscale spatial and spectral features. Third, we use a swin transformer to extract high-level multiscale features. Moreover, it is not easy to obtain satisfactory results using only one type of feature. 

The main contributions of this paper are as follows:

- Various deep multiscale feature learning models were identified with distinct feature extraction abilities, including an encoder–decoder for reconstruction features, CNNs for spatial features, and transformers for long-range or structural features.
- The proposed model combines and fuses these diverse multiscale features to create a comprehensive representation of hyperspectral images.
- We developed an effective weight fusion strategy to merge multiscale features, optimizing the integration process.
- We highlighted the model’s ability to capture and utilize valuable spatial–spectral information, leading to improved accuracy in classification results compared to existing approaches.

The rest of the paper is organized as follows: Section II explains the methods, Section III presents the dataset and experimental evaluation, Section IV shows the results, Section V provides the discussion, and Section VI concludes the paper. 

## 2. Methods

**Figure 1:**
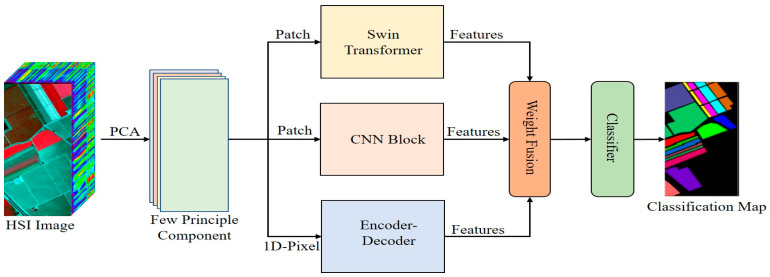
Proposed model illustration.

Figure 1 presents the proposed model. This section will explain the structure of the model and how the model works. In this study, we attempt to extract multiscale features from an HSI cube and consider different level feature extraction methods. Then, we develop a three-branch network for low and mid-range features using a CNN, for high-level features using a swin transformer, and for reconstruction features using an encoder–decoder.

(1) Swin Transformer

The pivotal disparities between the swin transformer and vision transformer (ViT) reside in their fundamental feature mapping strategies. ViT yields singular low-resolution feature maps due to its employment of a global self-attention mechanism, which consequently results in a quadratic computational complexity concerning input image dimension. On the other hand, swin transformers employ a novel approach of merging image patches to construct hierarchical feature maps, offering an ingenious solution that curtails the computational intricacy to a linear scale with respect to input image dimension. This approach utilizes local windows for self-attention computation, affording enhanced efficiency and scalability in processing images of varying sizes. The characteristics of the swin transformer make the model suitable for vision tasks such as image classification, image detection, and image segmentation. Swin transformers can extract multiscale features in the spatial dimension.

**Figure 2:**
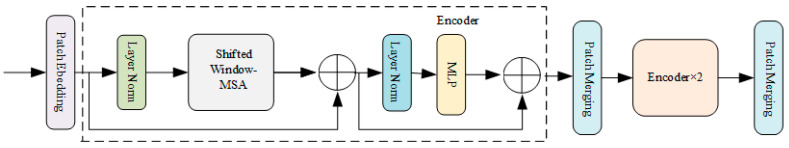
Illustration of swin transformer blocks.

The swin transformer model works by dividing the input image into non-overlapping patches. For the input of the model, first, we apply the dimensionality reduction technique to reduce the redundancy in data as well as computational complexity. In addition, without dimensionality reduction, overfitting occurs to alleviate the above problem; dimensionality reduction is important. $x={\mathbb{R}}^{m\;\times \;n\;\times \;b}$ is the input of the model, where m×n represents the height width of the HSI image and *b* represents the bands. y indicates the dimensionality reduction layer and y $\in {\mathbb{R}}^{z\;\times \;3}$**.** *z* represents the number of bands. After that, the patch token processes the input to the swin transformer. The model consists of shifted window self-attention, an MLP layer, and layer normalization layers. A patch-merging layer is adopted to reduce the number of tokens, and the model becomes deeper to generate spatial features. The core component of the swin transformer is shifted window self-attention. The shifted window technique is applied in image classification tasks. Figure 2 shows the blocks of the swin transformer.

**Figure 3:**
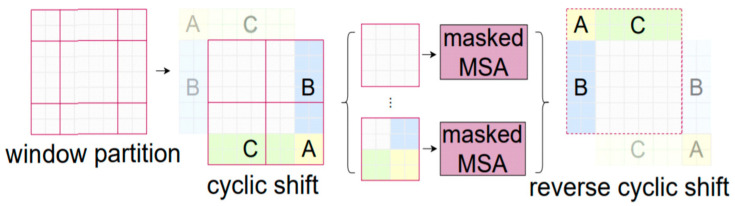
Illustration of shifted window partition in swin transformers [33].

The window partition process is computed as follows:

(1) $${\hat{z}}^{l}=WMSA\left(LN\left(z^{l\;-\;1}\right)\right)+z^{l\;-\;1},$$

(2) $$z^{l}=MLP\left(LN\left({\hat{z}}^{l}\right)\right)+{\hat{z}}^{l},$$

(3) $${\hat{z}}^{l+1}=SWMSA\left(LN\left(z^{l}\right)\right)+z^{l},$$

(4) $$z^{l+1}=MLP\left(LN\left({\hat{z}}^{l+1}\right)\right)+{\hat{z}}^{l+1},$$

where ${\hat{z}}^{l}$ represents the output of window multihead self-attention. $z^{l}$ represents the MLP output at the *l*th block. The process of window shifting shows in Figure 3.

(2) The convolutional Neural Network (CNN)

**Figure 4:**
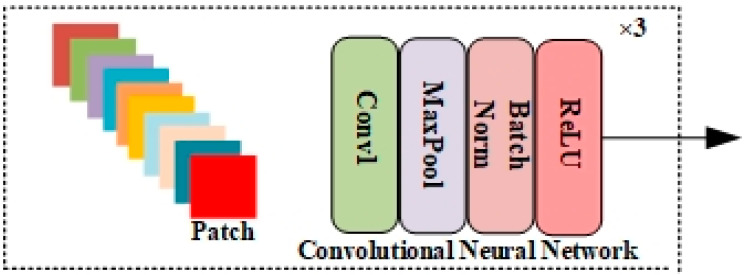
Illustration of convolutional neural block.

The second model is the CNN, which has achieved great success in the computer vision domain. It can extract multiscale spatial–spectral features simultaneously. This characteristic facilitates the differentiation of ground object materials in classification tasks. Let the input image cube be $X\in {\mathbb{R}}^{H\;\times \;W\;\times \;C}$, where H × W represents the height and width of the image and C represents the channels of the image. The block diagram of the CNN block is shown in Figure 4.

After using principle component analysis (PCA) to reduce the dimension of data to remove noise and redundancy, *c* is decreased to *D*. Input reduced data convert into small patches, and the process involves generating patches $D\in {\mathbb{R}}^{S\;\times \;S\;\times \;D}$ centered at the spatial location of (a, b), which covers spatial window size (*s* × *s*). Given *M* convolution kernel to input feature weights $w_{i}$, the output can be computed as

(5) $$Y=\delta \left(w_{i}*D\right),$$

where δ denotes the activation function. After the convolution layer is used to reduce the spatial size and extract more discriminative features, the maxpooling layer is used, where MP denotes the maxpool operation

(6) p=MP(Y).

After reduction, the spatial size batch normalization layer is used to normalize the incoming batches, which helps to train the model faster. After the normlization layer, an activation function is used called a rectified linear unit (ReLU) to introduce non-linearity to output neurons. One convolution block consists of a CNN layer, MXPOOL layer, batch normalization layer, and ReLU layer. In this paper, block three is set with a (8, 16, 32) filter size and one stride kernel size, 3 × 3, is used in all blocks.

(3) Encoder–Decoder (ED)

The third part of the model is the encoder–decoder, which is an unsupervised feature extraction method. Generally, the encoder–decoder is implemented in two ways: it is fully connected [34] and fully convolutional. In this paper, we choose a fully connected method with a band attention module (BAM). 

**Figure 5:**
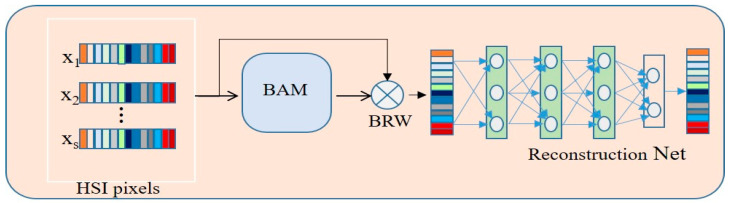
Illustration of the encoder–decoder.

The fundamental principle behind this type of encoder–decoder is to reconstruct the band information. This involves the retrieval of complete spectral details using a limited set of informative bands. Figure 1 shows that the overall architecture encompasses three key components: the band attention module (BAM), band reconstruction weights (BRW), and reconstruction network (RecNet). Figure 5 shows the internal process of encoder–decoder. 

The band attention module (BAM) is a function of *g.* Input *X* produces non-negative weights, and the tensor shape is $w\in {\mathbb{R}}^{1\;\times \;1\;\times \;b}$

(7) $$w=g\left(X;\theta_{b}\right),$$

where $\theta_{b}$ denotes the trainable parameter of BAM. To ensure the enforceable non-negativity of the acquired weights, the sigmoid function is incorporated into the output layer of the BAM module using following formulation:

(8) $$\phi \left(w\right)=\frac{1}{1+e^{-w}}.$$

In order to establish an interaction between the initial inputs and their corresponding weights, a band-wise multiplication operation, denoted as BRW, is operated. This operation can be succinctly described as follows:

(9) h=X⊗w.

Subsequently, we proceed with the utilization of RecNet to reconstruct the initial spectral band from its reweighted counterpart. Analogously, RecNet is characterized as a function denoted by *h*, which accepts a reweighted tensor *y* as input and produces its corresponding predictions.

(10) $$\hat{X}=h\left(y;\theta_{r}\right).$$

**Figure 6:**
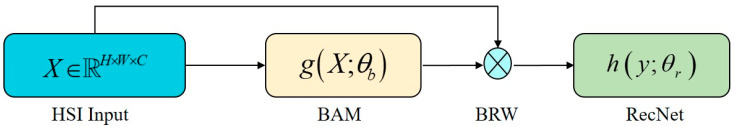
Block diagram of the encoder–decoder.

The reconstruction block simply consists of an MLP model with the same hidden neurons with a ReLU activation function for the reconstruction of features. Figure 6 shows the basic diagram of encoder decoder process.

(4) Weight Fusion

We discover three branch-extracted features from the swin transformer, CNN, and a fully connected encoder–decoder. These branches could present various features or characteristics of the data. The goal of the weight fusion technique is to provide each branch with an appropriate level of importance when classifying the result. First, we assess the importance of each branch. This could be based on the relevance of the information it captures. Once we determine the importance scores, we multiply each branch’s information by its corresponding weights. Fusing these features by summing operation, the formulation of weight fusion is calculated as:

(11) $$F_{1}=\lambda \times F^{CNN}+\left(1-\lambda \right)\times F^{ED},$$

where $F_{1}$ denotes two branches of CNN and ED fusion features and λ denotes the weighted parameter range in between [0, 1].

(12) $$F_{2}=\lambda \times F_{1}+\left(1-\lambda \right)\times F^{Transfromer},$$

where $F_{2}$ is the final output after fusing three branch features. Again, $F_{1}$ can multiply with transformer branch features.

(5) Classifier

**Figure 7:**
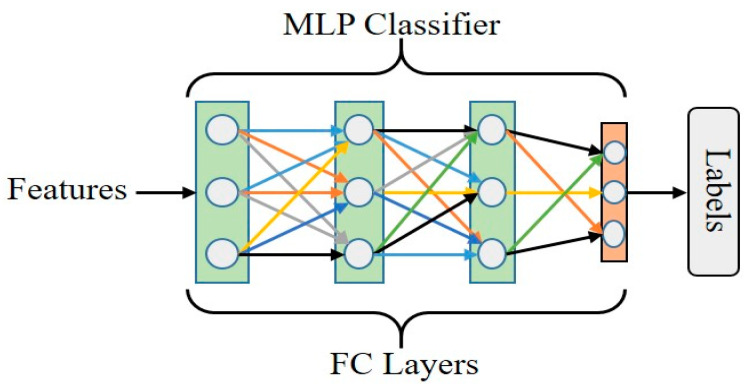
Illustration of classifier.

Figure 7 shows the multilayer perceptron classifier, a neural network model. It consists of multilayers of interconnected nodes, where each node in a layer is connected to all nodes in the subsequent layers. 

The input of the MLP classifier is multiscale features received from the weight fusion block; each node corresponds to specific weighted features and calculates the weighted sum. This weighted sum is then passed through an activation function ReLU. The activation function helps the network learn complex relationships within data. The second fully connected layer performs linear transformation, and the applied softmax function converts the output value into probabilities representing the likelihood of each class. The classes with the highest probability are predicted as the final classification.

In summary, we use the preprocessing technique principle component analysis (PCA) to reduce the noise and redundancy in data. After preprocessing, the input image data are embeded into three different branches: the first is a swin transformer to extract high-level features, the second is a convolutional neural network to extract low-level features, and the third is an encoder–decoder with a fully connected layer to extract reconstruction features from hyperspectral data. The details of working all branches is described in sections. After extracting multiscale features, the weight fusion technique is applied to fuse features, computational complexity is very low. Moreover, the classifier is used to classify the image.

## 3. Dataset and Experimental Evaluation

### 3.1. Dataset

In this paper, the performance of the model is evaluated using three widely used hyperspectral datasets, including the Xuzhou dataset, the WHU-Hi-LongKou dataset, and the Salinas dataset. The details regarding the three datasets can be found in Table 1, Table 2 and Table 3.

The Xuzhou dataset has false color and ground truth. This dataset was acquired by a HYPEX spectral camera over the Xuzhou peri-urban site. The spatial resolution of this dataset is a pixel with a high resolution of 0.73 m/pixel. The spectrum used in this dataset is 436 after the removal of noisy bands from 415 nm to 825 nm. The application of the Xuzhou dataset can be employed in mineral classification.

The WHU-Hi-LongKou dataset was acquired by a Nano-Hyperspec imaging sensor in Longkou Town, Hubei Province, China. The size of the imagery is 550 × 400 pixels. There are 270 bands from 400 to 1000 nm, and the spatial resolution of the UAV-borne hyperspectral imagery is approximately 0.463 m. The WHU-Hi-LongKou dataset is used for fine crop classification.

The other dataset we used for this experiment is Salinas, which was acquired by a (AVIRIS) sensor. A spatial dimension of 512 × 217 with 3.7 m spatial resolution was used. This dataset comprises 224 bands; however, 20 bands sensitive to water absorption were excluded, resulting in 204 bands used for experiments. There are 54,128 labeled pixels in total, spanning 16 different land-cover categories. Figure 5 shows the false color and ground truth of this dataset.

### 3.2. Experimental Evaluation

To obtain the quantitative performance of the proposed model, the classification results are evaluated in terms of three matrices: overall accuracy, average accuracy, and Kappa coefficient. The formulation of these matrices is as follows:

Overall accuracy (OA), which computes the *Number of Correct Pixels* over the number of overall samples

(13) $$OA=\frac{Number\;of\;Correctly\;Pixel}{Overall\;pixels}.$$

Average accuracy (AA) is a crucial assessment metric [36] that offers an insightful evaluation of classification proficiency. This metric computes the average accuracy achieved across all categories:

(14) $$AA=\frac{1}{n}\sum_{i=1}^{n}x_{i},$$

where *n* is the number of classes, and *x* is a correctly assigned pixel to a single class. In addition, the Kappa coefficient is calculated as follows [37]:

(15) $$Kappa=\frac{OA-p_{e}}{1-p_{e}},$$

where Kappa determines the agreement between the predicted classification map and ground truth map. $p_{e}$ represents the expected agreement between the model classification map and ground truth maps by chance probability. Usually, Kappa ≥ 80 indicates good agreement, while Kappa ≤ 0.4 indicates poor performance of the model.

### 3.3. Experimental Setting

In this experiment, we set the Adam optimizer while categorical cross entropy was chosen as a loss function to train the proposed model. The learning rate was set 0.001 and weight decay to 0.0001. For training and testing samples, we set 1% for training on the Xuzhou and Salinas datasets 99% to test the model. For the LK Dataset, we set 0.005% samples to train and 99.95% to test the model. We set the batch size, and the epoch was 64, 100 for simulation. All the experiments were run on NVIDIA RTX 3060 GPU with 64 GB RAM. We chose PYTHON 3.8 with a Tensorflow library. For the swin transformer, we set patch size as 4 × 4. When the spatial size could not be evenly divided, zero padding was applied. We also set three channels after dimensionality reduction. The window size was set to default seven.

### 3.4. Model Parameters Selection

In this section, we discuss some parameters that impact classification accuracy, like principle component analysis (PCA), and the different number of patch sizes or window sizes.

(1) Impact of principle component analysis (PCA).

**Figure 8:**
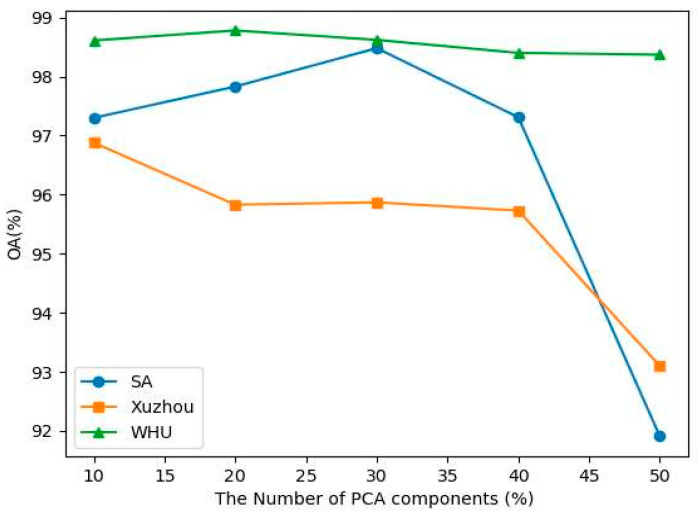
OA classification result (%) at different PCA components.

Extracting spectral information from bands is a very challenging task due to redundant and noisy data. In addition, it is very expensive with computational cost. Researchers found that reducing the dimensionality is the best way to extract the information from bands. To mitigate the aforementioned issue, we conducted comparative experiments on three datasets using 10, 20, 30, 40, and 50 PCA components and found from which band we should extract optimal information. Figure 8 shows the overall accuracy result on different PCA components.

(2) Impact of Patch size

**Figure 9:**
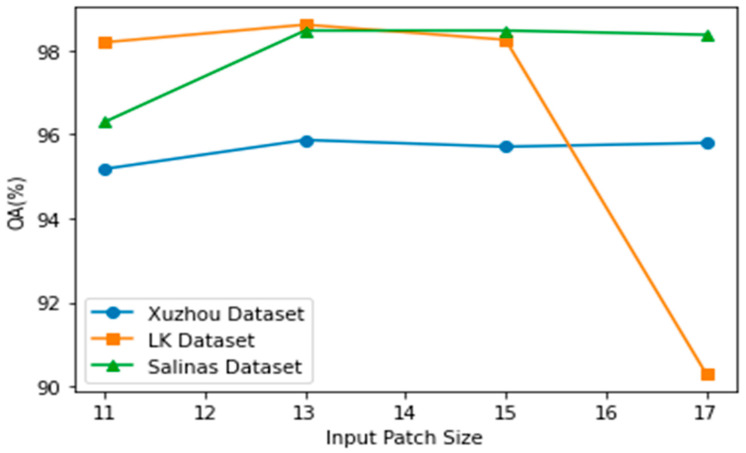
OA classification result (%) and different patch sizes.

Different input patch sizes affect classification accuracy, so a selection of input patch size is important. Figure 9 shows the overall accuracy result on different patch sizes. We conducted experiments on all datasets to determine the optimal size of input. In this paper, we chose the optimal patch size as 13 × 13 for the input size of the model. We found that when increasing the patch size at some points, the accuracy stopped increasing.

## 4. Results

To compare the classification results of the proposed model, comparative experiments were conducted and the models used were SVM [37], 2D-CNN [38], 3D-CNN [39], Hybrid [22], DFFN [41], Bam-CM [42], ViT [26], SwinT [33], SSFTT [30], and CT Mixer [31].

The comparison classification result of the Xuzhou Dataset is shown in Table 4. One can see that the quantitative result of the SVM-based method obtained 84.39% classification accuracy compared to a single deep model with fewer parameters. This means traditional classification methods still have some advantages in specific cases. On the other hand, as one can see, OA of state-of-the-art methods 3D, Hybrid, ViT, SwinT, and CT Mixer was 94.41%, 95.09%, 92.50%, and 95.72%, respectively; on the other hand, our proposed model obtained 95.87% classification accuracy on the Xuzhou dataset with 1% training samples. 

Additionally, the observation reflects the effectiveness of the proposed model in seamlessly integrating features extracted from different models. As a result, it significantly enhances OA classification performance. 

**Figure 10:**
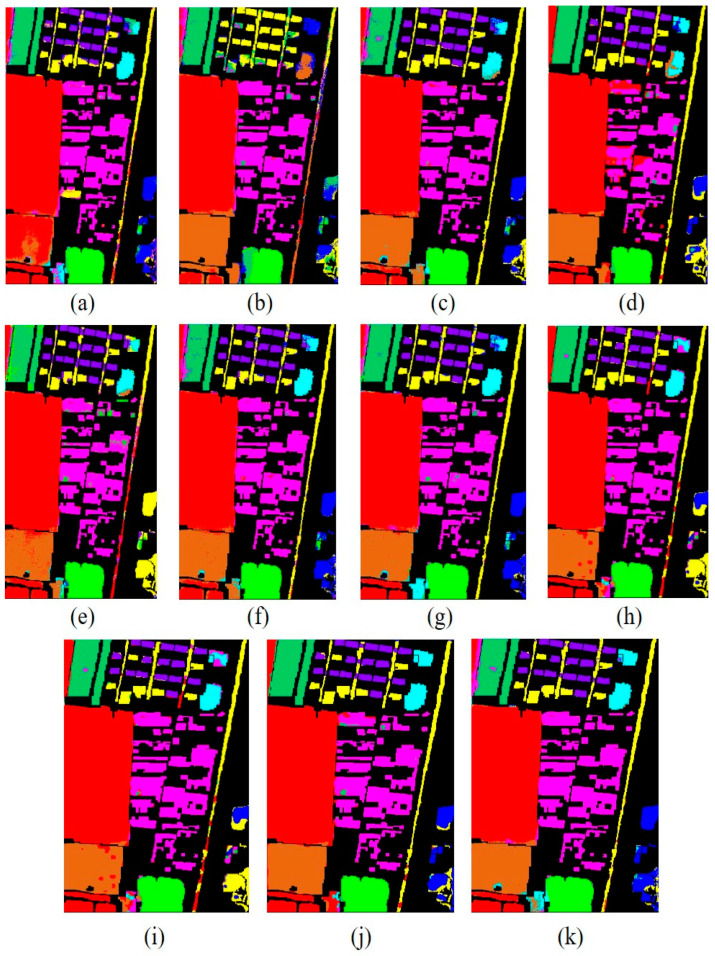
Classification maps obtained by different methods for the Xuzhou dataset. (**a**) SVM, (**b**) 2D, (**c**) 3D, (**d**) Hybrid, (**e**) DFFN, (**f**) Bam-CM, (**g**) ViT, (**h**) ST, (**i**) CT Mixer, (**j**) SSFTT, and (**k**) proposed.

Figure 10 shows the classification maps of different methods on the Xuzhou dataset. As shown in the figure, more training samples and more model layers can obtain better results with less noise. As one can see, ViT obtains excellent classification accuracy with less noise and intra-class smoothness. In addition, the proposed model not only obtains multiscale spectral–spatial features but also includes high semantic features from the transformer and reconstruction features from the encoder–decoder. It can obtain a better classification map and obtain more information that is detailed. The comparison classification result of the Salinas dataset is shown in Table 5. From the table, one can see first the shallow traditional multiscale feature learning method is lower than the deep learning model in terms of OA, AA, and Kappa coefficient. In contrast, 2D achieved the best result due to its spatial feature extraction capability. On the other hand, the proposed model is better than other multiscale feature learning models in terms of OA. Furthermore, SSFTT and CT Mixer performed better than other deep models. Additionally, our proposed model achieved better multiscale features among other single models and obtained the highest classification accuracy in some categories. SSFTT and CT Mixer use combined CNN and transformer models for feature extraction; the combination of both models is the best choice for local and global information. 

**Figure 11:**
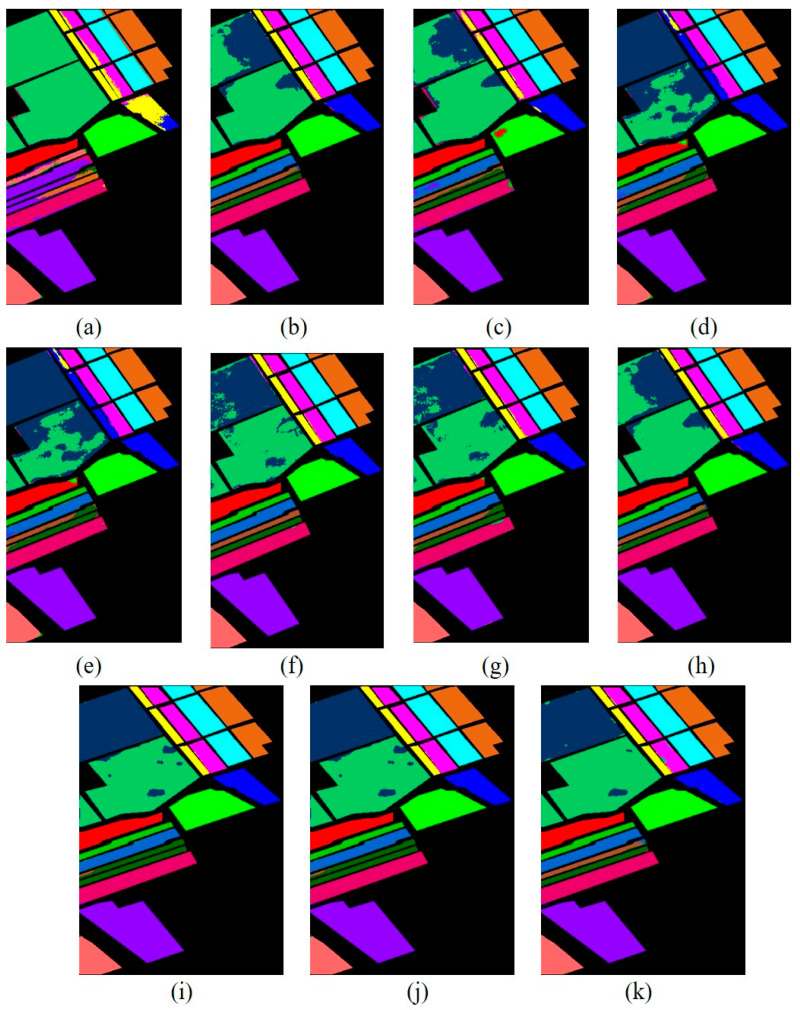
Classification maps of different methods for Salinas dataset. (**a**) SVM, (**b**) 2D, (**c**) 3D, (**d**) Hybrid, (**e**) DFFN, (**f**) Bam-CM, (**g**) ViT, (**h**) ST, (**i**) CT Mixer, (**j**) SSFTT, and (**k**) proposed.

Figure 11 shows the classification maps of different methods; as we can see, the SSFTT model and proposed model have less noise. Our classification is almost near to the ground truth image. As the level of noise increases, the accuracy of the classification maps tends to decrease. This observation highlights the importance of noise reduction techniques and the need to address and minimize noise effects to improve the accuracy of classification results. As one can see, the Xuzhou dataset and the proposed model improve 12.48%, 15.31%, 2.67%, 2.46%, 5.8%, 7.38%, 4.37%, 3.47%, and 1.15% OA accuracy compared to SVM, 2D-CNN, 3D-CNN, Hybrid, DFFN, Bam-CM, Vit, SwinT, SSFTT, and CT mixer. Traditional methods are not able to fully extract multiscale spectral–spatial features. The 2D-CNN has the ability to extract both spectral and spatial features, but for semantic features, in terms of semantic or high level features, CNNs are not able to extract these types of features. The transformer model has the ability to learn sematic features; however, a CNN and transformer combined can extract more discriminative features like CT Mixer in terms of improved classification accuracy. The comparison results of the WHU-Hi-LongKou dataset are shown in Table 6 with different methods. In this experiment, one can see that the traditional method of SVM performed better than other deep models. Due to some class variability, the 2D-CNN network also performed well due to its spatial feature extraction capability. From the other state-of-the-art methods, vision transformer also performed well to extract long-range features. On the other hand, our proposed model performed significantly well in terms of OA, AA, and Kappa coefficient.

**Figure 12:**
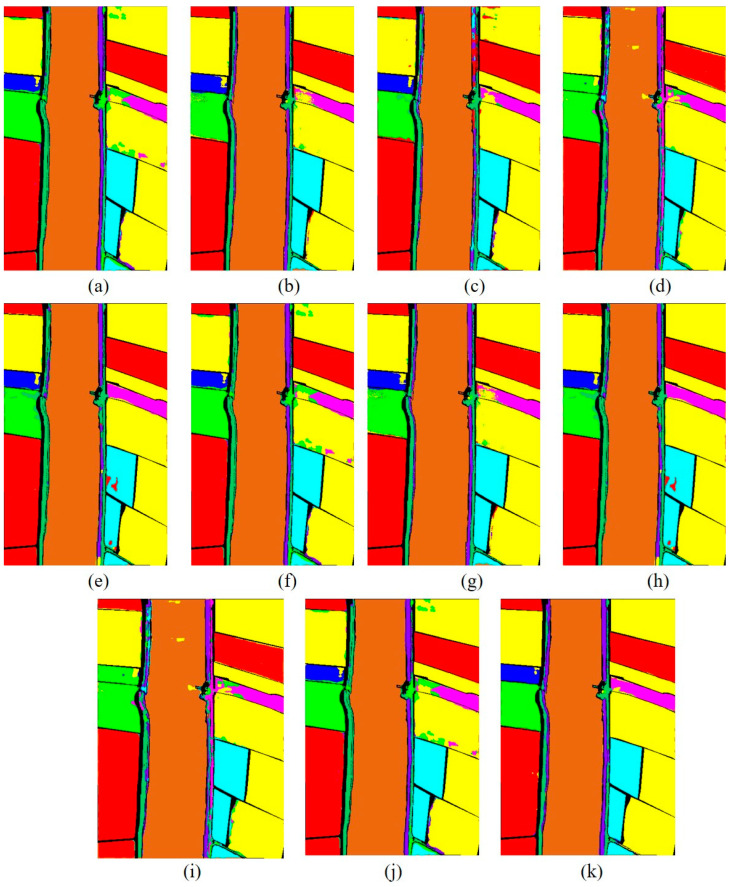
Classification map of different methods. (**a**) SVM, (**b**) 2D, (**c**) 3D, (**d**) Hybrid, (**e**) DFFN, (**f**) Bam-CM, (**g**) ViT, (**h**) ST, (**i**) CT Mixer, (**j**) SSFTT, and (**k**) proposed.

Figure 12 shows the classification maps of different methods. As one can see, SVM, 2DCNN, ViT, and our proposed model have competitive classification results from classification maps; therefore, there is less noise in the maps. Our proposed method can achieve high accuracy in some classes. If the number of samples is increased, the deep model layers model can achieve higher accuracy due to the computational complexity increase. Our proposed model shows impressive classification performance due to the best weight fusion design strategy. The weight fusion strategy makes full use of multiscale feature fusion from different branches. The reconstruction feature from the encoder–decoder model can enhance the fused feature more. Our proposed model systematically combined multiscale features. It can be seen from Table 4, Table 5 and Table 6 that our proposed model obtained better results than other state-of-the-art methods.

## 5. Discussion

This paper developed a three-branch unified model to extract multiscale features. Hyperspectral image data have different kinds of features such as texture, structural features of data, and land cover object shape and size. Due to different model characterization, we used three different deep learning models to extract multiscale features at different levels and fused these multiscale features with weight fusion techniques to provide different features with different weights. It can be seen from Table 4, Table 5 and Table 6 that the classical method SVM, several deep learning methods, 2D, 3D, Hybrid, DFFN, and Bam-CM, and recently introduced vision transformer-based methods, ViT, Swin Transformer, SSFTT, and CT Mixer, were considered for comparison.

Our experiment shows that the proposed methods achieve the best classification results in terms of overall accuracy (OA), average accuracy (AA), and Kappa coefficient (k) on the three publically available datasets. Taking the example of the WHU-Hi-LonhKou dataset, Hybrid and SSFTT models are not able to extract class C3 features. For class C4 and class C5, our proposed method achieved satisfactory results. The strength of our model in all classes except class C9 achieved more than 90% accuracy, which exhibits the robustness and discriminative power of the model. Compared with the CNN model and transformer model, the OA of the proposed method improved 0.91%, 4.84%, 3.66%, and 2.72% on the LK dataset with DFFN, Bam-CM, SSFTT, and CT Mixer models, respectively. The proposed model combines the advantage of each branch to extract different kinds of multiscale features at each level to improve the classification performance. Moreover, the proposed method adopts a comprehensive feature fusion technique that could potentially improve the model’s capability to extract multiscale information. In addition to the other deep feature fusion models, such as DeepCervix, multiscale feature learning methods cannot achieve semantic high-level features; also, they use concatenation and add methods to fuse multiscale features. These fusion techniques might introduce redundant information, especially if the branches capture similar multiscale features. Because of imbalance and small training samples, these models are susceptible to overfitting. On the other hand, our proposed model achieved satisfactory classification results on small training samples. The weight fusion technique facilitates the integration of multiscale features extracted by different modules. This fusion strategy can lead to more comprehensive and refined multiscale feature representation. By integrating multiple modules and fusion techniques, the model offers a holistic approach to hyperspectral image classification, potentially improving its ability to handle complex real-world scenarios. In terms of the feature fusion technique, weight fusion allows the model to assign different importance to features from different branches, emphasizing more relevant features; furthermore, weight fusion technique controls the dimensionality and reduces the computational cost.

### 5.1. Ablation Study

To highlight the effectiveness of the proposed model, an investigation was conducted on the Xuzhou dataset to examine the impact of different combinations of network components. The findings are presented in Table 7; the result demonstrates that our fusion technique, as proposed, achieves superior performance when compared to other combination approaches.

### 5.2. Different Models on Different Training Samples over the Xuzhou Dataset

**Figure 13:**
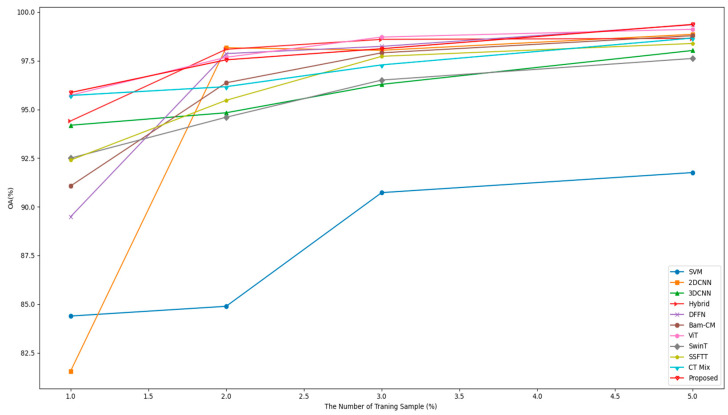
OA 1–5% of the training sample on the Xuzhou dataset.

Model performance can be effectively evaluated by examining the classification accuracy across different numbers of training samples. To access this, we randomly selected 1%, 2%, 3%, and 5% data for training and the remaining for testing the model. Figure 13 shows that the classification accuracy increases when the number of training samples increases. The strategy shows that the proposed model is always effective on a small number of training samples.

## 6. Conclusions

In this paper, we proposed a deep learning model based on a parallel branch structure for hyperspectral image classification tasks. The model can mine multiscale features such as reconstructive spectral features and low-level and high-level features and perform a weight fusion strategy. Due to the high dimensionality of the data, we used the PCA algorithm to reduce data redundancy. Our experimental results show that the proposed model performs well in terms of OA, AA, and Kappa coefficient with a small number of training samples. The proposed model has fewer parameters than other multiscale feature learning models. In future endeavors, we aim to explore the utilization of lighter architecture with fewer training samples. 

## Figures and Tables

**Table 1:** Xuzhou dataset labeled samples.

No	Class	Color	Train	Samples	False Color	Ground Truth
C1	BareLand-1		263	26,396	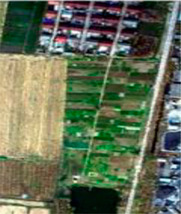	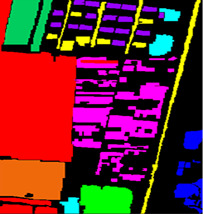
C2	Lakes		40	4027		
C3	Coals		27	2783		
C4	Cement		52	5214		
C5	Crops-1		131	13,184		
C6	Trees		24	2436		
C7	Bareland-2		70	6990		
C8	Crops-2		47	4777		
C9	Red tiles		30	3070		
Total			684	68,877		

**Table 2:** WHU-Hi-LongKou dataset labeled samples.

No	Class	Color	Train	Samples	False Color	Ground Truth
C1	Corn		172	34,511	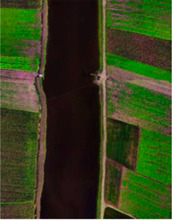	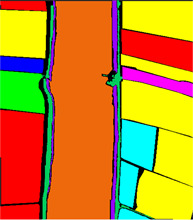
C2	Cotton		41	8374		
C3	Sesame		15	3031		
C4	Broad-leaf soybean		316	63,212		
C5	Narrow-leaf soybean		20	4151		
C6	Rice		59	11,854		
C7	Water		335	67,056		
C8	Road and house		35	7124		
C9	Mixed weeds		26	5229		
Total			1019	204,542		

**Table 3:** Salinas dataset labeled samples.

No	Class	Color	Train	Samples	False Color	Ground Truth
C1	Broccoli-g-w-1		20	2009	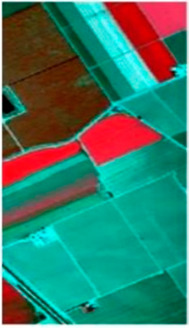	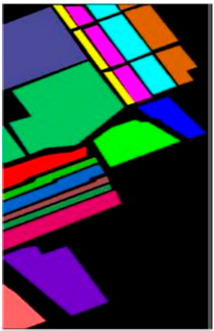
C2	Broccoli-g-gw-2		37	3726		
C3	Fallow		19	1976		
C4	Fallow-rough-plow		13	1394		
C5	Fallow-smooth		26	2678		
C6	Stuble		39	3959		
C7	Cerely		35	3579		
C8	Graphes-untrained		112	11,271		
C9	Soil-vineyard-develop		62	6203		
C10	Corn-senesced-g-w		32	3278		
C11	Lettuce-romaine-4 kw		10	1068		
C12	Lettuce-romaine-5 kw		19	1927		
C13	Lettuce-romaine-6 kw		9	916		
C14	Lettuce-romaine-7 kw		10	1070		
C15	Vineyard_untrained		72	7268		
C16	Vinyard_verticle_trellis		18	1807		
Total			533	54,129		

**Table 4:** Classification result (%) on the Xuzhou dataset with 1% samples.

Class ID	SVM	2D	3D	Hybrid	DFFN	Bam-CM	VIT	SWIN	SSFTT	CT MIX	Proposed
C1	92.41	77.04	96.96	94.52	90.80	96.52	94.36	96.95	98.07	97.97	96.61
C2	88.15	94.95	98.19	96.48	97.86	97.36	95.24	94.45	96.16	98.79	97.34
C3	6.32	90.23	86.38	91.72	53.75	83.52	89.83	82.57	45.15	76.58	**93.01**
C4	86.04	73.28	96.55	91.88	99.07	85.76	94.78	75.26	88.19	93.70	93.43
C5	87.64	81.79	99.56	93.51	100	93.02	98.28	94.33	98.74	96.30	96.75
C6	58.29	84.57	8.87	92.66	68.32	37.60	87.84	84.61	70.27	75.70	**88.68**
C7	76.10	79.36	99.34	95.80	100	89.69	95.16	92.60	94.37	97.28	98.15
C8	79.33	91.28	97.48	99.36	56.98	87.56	98.42	93.74	97.50	100	97.50
C9	77.84	95.65	96.15	91.83	81.67	94.43	97.30	86.17	93.61	96.38	95.50
**OA**	84.39	81.56	94.19	94.41	89.49	91.07	94.73	92.50	93.40	95.72	**96.87**
**AA**	72.45	85.35	86.61	94.20	83.16	85.05	93.36	90.44	91.56	92.52	**94.66**
**Kappa**	80.05	77.57	92.62	92.95	86.70	88.64	93.12	88.96	90.26	94.55	**95.76**

**Table 5:** Classification result (%) on the Salinas dataset with 1% samples.

Class ID	SVM	2D	3D	Hybrid	DFFN	Bam-CM	VIT	SWIN	SSFTT	CT MIX	Proposed
C1	92.13	99.79	95.47	98.13	99.49	98.49	99.34	53.79	100	99.94	98.84
C2	94.40	100	99.24	100	99.91	99.70	100	98.37	100	100	**100**
C3	54.66	99.69	98.77	99.33	99.79	97.39	99.74	84.15	99.18	100	99.94
C4	97.48	92.17	98.69	96.44	74.78	5.65	100	99.49	99.92	99.42	98.95
C5	82.75	97.01	82.57	98.07	100	93.54	96.60	98.37	99.24	99.66	99.81
C6	99.51	100	100	100	99.51	98.49	99.97	100	100	100	**100**
C7	90.66	100	99.57	99.91	99.91	99.15	99.85	96.58	99.94	100	99.15
C8	57.68	92.89	80.86	98.03	100	88.69	89.41	89.71	96.51	94.41	96.45
C9	86.77	100	99.67	100	99.88	99.67	99.90	99.96	100	100	**100**
C10	65.30	97.19	79.93	95.00	96.51	85.11	93.77	96.91	99.13	99.22	98.90
C11	20.10	99.62	98.95	98.77	100	44.27	100	83.63	99.05	100	99.43
C12	93.73	99.84	92.13	100	65.25	75.62	98.53	97.69	99.73	98.74	99.94
C13	15.20	24.80	75.52	57.99	32.41	42.22	79.16	96.58	95.58	6.72	**99.00**
C14	91.36	99.52	74.12	90.17	96.60	70.91	96.69	97.45	62.13	99.71	98.67
C15	58.23	67.94	83.11	85.15	25.86	65.81	87.88	84.82	98.69	99.97	98.98
C16	18.30	99.16	97.31	99.44	99.60	87.75	100	93.46	99.94	99.55	**100**
**OA**	73.47	92.35	89.99	96.06	86.63	85.10	95.09	92.17	98.12	97.10	**98.48**
**AA**	69.89	91.85	90.99	94.78	86.84	78.28	96.30	91.93	96.81	93.58	**98.94**
**Kappa**	70.25	91.46	89.92	95.60	84.96	83.32	94.54	91.27	97.90	96.78	**98.31**

**Table 6:** Classification result (%) on the WHU-Hi-LongKou dataset with 0.005% samples.

Class ID	SVM	2D	3D	Hybrid	DFFN	Bam-CM	VIT	SWIN	SSFTT	CT MIX	Proposed
C1	98.20	99.96	99.55	99.88	99.93	99.35	99.94	99.86	97.05	99.48	99.91
C2	82.08	93.90	78.08	97.56	99.74	75.67	90.83	76.10	97.77	99.81	96.65
C3	80.02	85.41	83.72	2.18	96.75	30.13	90.51	95.09	1.39	86.04	92.24
C4	95.45	99.51	98.40	98.63	98.19	98.72	98.99	97.10	98.66	96.20	**99.65**
C5	64.81	67.62	71.50	80.36	60.55	39.22	75.18	86.31	78.66	67.40	**90.00**
C6	97.99	98.72	99.50	91.72	98.70	92.71	98.32	98.44	98.97	96.63	97.97
C7	98.55	100	99.66	99.87	98.61	99.90	99.99	99.89	98.97	99.50	99.99
C8	89.84	93.10	89.00	97.43	97.27	77.10	96.81	92.46	55.01	76.00	93.58
C9	83.45	86.89	86.00	68.87	90.90	53.08	88.83	87.79	72.23	69.88	82.08
**OA**	95.83	98.07	96.83	95.61	97.71	93.78	98.16	97.05	94.96	95.90	**98.62**
**AA**	87.81	91.68	89.49	85.28	93.41	73.99	93.27	92.56	85.86	87.88	**94.68**
**Kappa**	94.49	97.46	95.81	94.22	97.00	91.70	97.58	96.13	92.06	94.63	**98.15**

**Table 7:** OA (%) result of ablation study of different combinations of a model over the Xuzhou dataset.

Methods	Encoder-Decoder	CNN	SwinT	ED + CNN	ED + SwinT	CNN + SwinT	Proposed
**OA (%)**	88.90	92.93	93.00	94.00	93.65	94.78	**96.87**
**AA (%)**	85.27	92.91	89.49	92.79	91.96	92.75	**94.66**
**Kappa (%)**	85.89	91.14	91.10	92.45	92.00	93.38	**95.76**

## Data Availability

The data can be obtained from: http://www.ehu.eus/ccwintco/index.php?title=Hyperspectral_Remote_Sensing_Scenes (accessed on 12 September 2022).
